# White matter integrity in delinquent emerging adults and non-delinquent controls, and its relationship with aggression, psychopathic traits, and cannabis use

**DOI:** 10.3389/fnbeh.2022.895798

**Published:** 2022-07-28

**Authors:** Jochem M. Jansen, Josjan Zijlmans, Arne Popma, Reshmi Marhe

**Affiliations:** ^1^Faculty of Law, Institute for Criminal Law and Criminology, Leiden University, Leiden, Netherlands; ^2^Department of Child and Adolescent Psychiatry and Psychosocial Care, Amsterdam University Medical Center, Amsterdam Public Health Research Institute, Vrije Universiteit Amsterdam, Amsterdam, Netherlands; ^3^Department of Psychology, Education and Child Studies, Erasmus School of Social and Behavioural Sciences, Erasmus University Rotterdam, Rotterdam, Netherlands

**Keywords:** delinquency, psychopathy, aggression, diffusion tensor imaging (DTI), substance use

## Abstract

**Background:**

Atypical white matter integrity may be one of the biological factors related to delinquency. In adults, decreased white matter integrity has been related to antisocial behavior, but findings from research in adolescent and young adult populations are either mixed or lacking. Here we investigated this association within a naturalistic sample of delinquent young adults (age 18–27).

**Methods:**

In a sample of 95 young adult, delinquent men and 22 age and gender matched controls, we assessed white matter integrity through fractional anisotropy and mean diffusivity measures. We compared white matter integrity between the groups, and within the delinquent group assessed the association between white matter integrity and aggression, psychopathic traits, and cannabis use.

**Results:**

We found no differences in fractional anisotropy or mean diffusivity between delinquent young adults and non-delinquent controls. Additionally, within the group of delinquent young adults, we found no associations between white matter integrity and aggression, psychopathic traits, or cannabis use.

**Conclusion:**

Our null findings suggest that white matter integrity differences may be unrelated to antisocial behavior in emerging adults, and/or that white matter differences between delinquent populations and controls may only arise later in life.

## Introduction

Emerging adulthood is defined as the transition from adolescence to adulthood and is considered a challenging and important developmental period, ranging from 18 to 29 years of age (Arnett, 2000). This period is characterized by rapid changes in social context, greater freedom, and less social control compared to adolescence (Henin and Berman, 2016). Recent studies show emerging adulthood is a relevant period regarding onset and changes in offending behavior, in part because the peak of the age-crime curve lays between the ages of 21 and 25 (Craig and Piquero, 2015).

Studies assessing normal brain development show that gray matter decreases and white matter increases may underly the reduction in risk taking and the increases in self-regulation, future orientation, and planning that take place during emerging adulthood (Taber-Thomas and Pérez-Edgar, 2015). Whereas, brain development is deemed crucial for the relationship between adolescence and crime (Steinberg, 2009), there is little research specifically investigating brain abnormalities related to criminal behavior in emerging adulthood.

A recent review on the relationship between white matter integrity and antisocial behavior (including aggression and psychopathic/callous-unemotional traits) across the lifespan underlines this limitation in the literature. Results show antisocial behavior is negatively associated with white matter integrity across a range of white matter tracts in adults, but that studies in adolescent populations are mixed and inconclusive, whereas studies in emerging adulthood are absent all together (Waller et al., 2017). These contradicting results could not be explained by differences in sample type, age, or analysis strategy, and future studies are advised to conduct studies in a narrower age range.

White matter integrity can be assessed with diffusion tensor imaging, measuring the displacement of water molecules within white matter tracts. The structure of the axonal cell membranes and myelin sheath hinder the diffusion of water molecules in other directions than along the fiber tract (Pierpaoli and Basser, 1996). Four often used measures for white matter integrity are fractional anisotropy (FA),mean diffusivity (MD), radial diffusivity (RD) and axial diffusivity (AD), reflecting diffusion of water molecules along the fiber tract (FA), overall diffusion (MD), average diffusivity along the other two minor axes (RD) and the magnitude of diffusion parallel to fiber tracts (AD) respectively (Beaulieu, 2002). Higher FA or AD and lower MD or RD values reflect better white matter integrity, associated with denser axonal packing, increased coherence of axon orientations, and greater myelination (Beaulieu, 2002).

The current study assesses differences in white matter integrity between a relatively large sample of delinquent young adults (age 18–28) and age-matched healthy controls. Within the sample of delinquent young adults, the relationships between white matter integrity, aggression, psychopathic traits, and cannabis use are assessed.

## Materials and methods

### Participants, data acquisition and data preprocessing

A total of *n* = 95 delinquent young adult men and *n* = 22 age- and gender-matched healthy controls [*T*_(117)_ = 0.64 *p* = 0.52) were included in this study. Delinquent youth were recruited during resocialisation programs or outpatient treatment, and matched controls were recruited from secondary vocational education [described extensively elsewhere; (Zijlmans et al., 2018)]. The Self-Sufficiency Matrix (Fassaert et al., 2014) was used to assess self-sufficiency and degree of multi-problem issues. Delinquent youth were not self-sufficient and had multi-problem issues—which was checked through chart review, and matched controls were self-sufficient and did not have multi-problem issues. Participants subsequently completed self-report assessments on aggression (Reactive Proactive Aggression Questionnaire; RPQ), psychopathic traits (Youth Psychopathy Inventory; YPI), and cannabis use (days used in last month), (see Table 1). Scans were visually inspected for quality control and tensor orientation. In each group, two participants were excluded from analyses due to MRI artifacts. Diffusion tensor imaging sequences were acquired using one gradient value of *b* = 0 and in 32 orthogonal directions with a *b*-value of 1,000 s/mm^2^. DTI data were pre-processed using standard procedures in FSL (FMRIB Software Library), and include skull stripping, eddy current correction, tensor fitting, transformation to standard space, and Tract-Based Spatial Statistics (see Supplementary Information).

**Table 1:** Participant characteristics.

	**Control**		**Delinquent**	
	**Mean**	**SD**	**Mean**	**SD**
Age	23.4	2.66	22.46	2.45
IQ	-	-	82.55	10.97
RPQ Total	11.59	8.2	16.86	7.99
RPQ Proactive aggression	8.23	5	11.61	4.73
RPQ Reactive Aggression	3.36	3.61	5.25	4.41
YPI Total	37.33	5.74	34.59	8.58
YPI Affective	11.43	3.23	10.9	3.73
YPI Behavioral	12.19	2.66	12.4	3.31
YPI Interpersonal	13.71	3.62	11.28	3.72
Cannabis (days used in last month)	3.96	6.08	14.81	13.39

*This table shows the participant characteristics for both the delinquent and control groups. RPQ, Reactive Proactive Aggression Questionnaire; YPI, Youth Psychopathy Inventory*.

Group comparisons between the delinquent and matched control group, as well as the within delinquent group regression analyses for aggression and psychopathic traits (including subscales) were carried out with permutation-based analysis (Nichols and Holmes, 2002), through Randomize implemented in FSL, utilizing threshold-free cluster-enhancement method (TFCE) (Smith and Nichols, 2009). Statistical maps were then thresholded at *P* < 0.05 using family-wiser error (FWE) correction. Analyses were not corrected for multiple testing.

This study was carried out in accordance with the recommendations of the Medical Ethical Committee of the VU University Medical Center. The protocol was approved by the VU University Medical Center Medical Ethical Committee (registration number 2013.422—NL46906.029.13). All subjects gave written informed consent in accordance with the Declaration of Helsinki. Participants received a reimbursement of 30 euros for their participation in the MRI protocol and an EEG protocol, which was administered on another day.

### Analyses

#### Questionnaires

We assessed differences in aggression, psychopathic traits, and cannabis use between groups with ANCOVAs in SPSS with (1) aggression (RPQ total score, and reactive and proactive aggression subscales), (2) psychopathic traits (YPI total score, affective, interpersonal, and behavioral components), and (3) cannabis use **(**days used in last month**)** as dependent variables, while correcting for age.

#### White matter integrity

Group differences in white matter integrity were assessed through permutation testing with Randomize in FSL, using fractional anisotropy (FA), mean diffusivity (MD), radial diffusivity (RD) and axial diffusivity (AD) as dependent variables, while correcting for age (Winkler et al., 2014).

Within the delinquent group, regression analyses were conducted through permutation testing with Randomize in FSL, assessing the relationship between FA,MD, RD and AD with (1) aggression (RPQ total score, and reactive and proactive aggression subscales), (2) psychopathic traits (YPI total score, and affective, interpersonal, and behavioral components), and (3) cannabis use, while correcting for age and IQ.

## Results

### Questionnaires

Results reveal that delinquent young adults do show higher self-reported levels of aggression [*F*_(1, 114)_ = 6.40 *p* = 0.01], but not psychopathic traits [*F*_(1, 112)_ = 2.50 *p* = 0.12]. Analysis of the questionnaire subscales reveals higher self-reported levels of proactive aggression [*F*_(1, 114)_ = 7.71 *P* < 0.01], interpersonal psychopathic traits [*F*_(1, 112)_ = 7.98 *P* < 0.01], and cannabis use [*F*_(1, 113)_ = 13.51 *P* < 0.001] in the delinquent group vs. the control group, but not higher reactive aggression [*F*_(1, 114)_ = 2.68 *p* = 0.10], affective psychopathic traits [*F*_(1, 112)_ = 0.52 *p* = 0.47], or behavioral psychopathic traits [*F*_(1, 112)_ = 0.01 *p* = 0.93].

### White matter integrity

Results of the white matter integrity analyses reveal no differences in fractional anisotropy, mean diffusivity, radial diffusivity or axial diffusivity between delinquent and non-delinquent controls. Additionally, within the group of delinquent young adults, fractional anisotropy, mean diffusivity, radial diffusivity nor axial diffusivity were related to aggression, psychopathic traits, or cannabis use.

## Discussion

The current study assessed possible differences between delinquent emerging adults and age-matched healthy controls in white matter integrity, but no group differences in FA, MD, RD or AD values were found. Even though differences in self-reported aggression—especially proactive aggression—cannabis use and interpersonal psychopathic traits were found between groups, these were not related to white matter integrity within the delinquent emerging adults. No differences were found for self-reported total, behavioral or affective psychopathic traits or reactive aggression.

Together with the inconsistent findings in the review by Waller et al. (2017), our results may suggest that (1) white matter integrity differences are unrelated to antisocial behavior in emerging adults, and/or that (2) white matter differences between delinquent populations and controls may only arise later in life. A later offset of white matter differences might be related to a number of factors, including known lifestyle differences between these groups [see Semenza (2018)]. Studies show that poor diet, lack of exercise, and drug/alcohol use decrease white matter integrity (Gow et al., 2012; Kokubun et al., 2020). Previous studies in substance dependent adolescents, however, indicate that white matter integrity is affected early on (Baker et al., 2013), contradicting the above explanation. Little is known about the effect of these lifestyle choices on white matter integrity in emerging adulthood—most studies were conducted in elderly subjects. Whether such lifestyle choices would mediate the relationship between delinquency and white matter integrity differences found in adults is an interesting topic for further research.

Another explanation for a delayed onset of white matter differences is the higher risk of traumatic brain injury (TBI) in delinquent populations (Durand et al., 2017). TBI is known to affect white matter integrity (Croall et al., 2014), may be present in about 46% of delinquent adults, and may be a risk factor for crime (Williams et al., 2018). TBI in delinquent populations is most often acquired due to traffic accidents and interpersonal violence (Durand et al., 2017), both of which are likely to peak during emerging adulthood (Craig and Piquero, 2015). Unfortunately, no data on TBI were collected for our current sample.

Surprisingly, the delinquent group and the matched controls did not differ on the affective and behavioral components of the YPI, nor on self-reported reactive aggression. This could be a chance finding, which could hinder interpretation of our group comparisons—but not the regression analysis for the delinquent sample. However, analysis of internal validity, construct validity and a factor analysis revealed that the YPI is a valid instrument in our sample [see Zijlmans et al. (2019) and Supplementary Information 2].

In addition to the points outlined above, delinquent young adults are often characterized by a multitude of problems, including addiction, mental health issues, and a poor social network (Zijlmans et al., 2021). Although our sample size of *n* = 22 should provide enough power to identify group differences in white matter integrity between the delinquent group and matched controls (De Santis et al., 2014), the heterogeneity within the delinquent group could be associated with variation in underlying white matter differences in location and integrity. This could obscure significant results from a group analysis. Future studies should investigate this variation by taking these multiple problems into account in group analyses, analyzing more heterogenous subgroups and using larger samples for sufficient power, while also increasing the number of controls. Assessing which lifestyle choices may mediate the relationship between white matter integrity and antisocial behavior in delinquent populations could also add to this growing body of research.

## Data availability statement

The raw data supporting the conclusions of this article will be made available by the authors, without undue reservation.

## Ethics statement

The studies involving human participants were reviewed and approved by Medical Ethical Committee of the Amsterdam University Medical Centers. The patients/participants provided their written informed consent to participate in this study.

## Author contributions

JZ and RM collected the data. JJ performed the statistical analysis and wrote the first draft of the manuscript. JJ and JZ wrote sections of the manuscript. All authors contributed to manuscript revision, read, and approved the submitted version. All authors contributed to the conception and design of the study.

## Funding

This research project is funded by the De Verre Bergen Foundation. De Verre Bergen Foundation is a venture philanthropy organization that aims to build a better Rotterdam through substantial investments in innovative, impactful social ventures. The financer was not involved in the design of the study nor the drafting of the manuscript. Furthermore, the financer was not involved in the process of data collection, analysis, and interpretation.

## Conflict of interest

The authors declare that the research was conducted in the absence of any commercial or financial relationships that could be construed as a potential conflict of interest.

## Publisher's note

All claims expressed in this article are solely those of the authors and do not necessarily represent those of their affiliated organizations, or those of the publisher, the editors and the reviewers. Any product that may be evaluated in this article, or claim that may be made by its manufacturer, is not guaranteed or endorsed by the publisher.
