# Supplementary material for: White matter integrity in delinquent emerging adults and non-delinquent controls, and its relationship with aggression, psychopathic traits, and cannabis use
Source: Front Behav Neurosci. 2022 Jul 28;16:895798. doi: 10.3389/fnbeh.2022.895798 (PMC9365978; doi:10.3389/fnbeh.2022.895798)
Supplement: Supplementary file 1 [file Data_Sheet_1.docx]

**Supplementary Information**

1. **DTI quality control and preprocessing**

Raw data was converted to 4D-nifti format, eddy current corrections were performed using FSL (FMRIB Software Library) and brain extraction was performed using the Brain Extraction Tool (BET ([Smith, 2002](#_ENREF_4))). Subsequently tensor fitting was performed using DTIfit, and all images were registered to a standardized MNI-152 space. Next, a mean FA image was created subsequently narrowing the tracts to create a mean FA skeleton representing the center of all tracts common to all subjects using Tract Based Spatial Statistics (TBSS ([Smith et al., 2006](#_ENREF_5))). In order to ensured that each subject’s skeleton was in the group space, yet represented the center of that subject’s own unique fiber tracts, the area around the skeleton in each subjects’ aligned FA map was searched and the highest local FA value was assigned to the skeleton. These steps were repeated for MD, RD, and AD maps. Finally, group comparisons and within group regression analyses were carried out with permutation-based analysis ([Nichols & Holmes, 2002](#_ENREF_2)), through Randomise implemented in FSL, utilizing threshold-free cluster-enhancement method (TFCE) ([Smith & Nichols, 2009](#_ENREF_6)). Statistical maps were then thresholded at P<.05 using family-wiser error (FWE) correction. Analyses were not corrected for multiple testing.

1. **Validity of YPI**

Unexpectedly, the multi-problem young adults scored slightly lower on the grandiose-manipulative interpersonal trait of psychopathy than the controls (M=11.33 vs. 13.20), whereas they scored similar to the controls on the affective callous-unemotional trait (M=10.74 vs. 11.56), the impulsive-irresponsible behavioral trait (M=12.21 vs. 11.88), and the total score (M=34.28 vs. 36.64; see Table 1). Possibly, this is a chance finding that has no consequences for our analysis as we are interested in psychopathic traits within the experimental group. Nonetheless, to ensure the validity of the YPI in our sample, we investigated internal validity by checking Cronbach’s alpha for each subscale. For the interpersonal factor α = 0.76, for the affective factor α=.65, for the behavioral factor α=.70, and for the total score α=.80. These figures are consistent with the literature ([Colins, Andershed, & Pardini, 2015](#_ENREF_1)). Moreover, to ensure criterion validity of the YPI in our sample we performed correlation analyses between the YPI subscales and relevant external criterium constructs: reactive aggression, proactive aggression, current cannabis use, and lifetime cannabis use. For the interpersonal factor we found positive correlations with both proactive (r = 0.20, p<0.05) and reactive (r = 0.39, p < .001) aggression. For the affective factor we found positive correlations with both proactive (r=.19, p < .05) and reactive (r = 0.30, p<0.01) aggression. For the behavioral factor we found positive correlations with both proactive (r=.42, p < .001) and reactive (r=.44, p<0.001) aggression, as well as recent (r =0.20, p<0.05) and lifetime (r = 0.20, p<0.05) cannabis use. Finally, we performed a confirmatory factor analysis on the three-factor structure of the YPI using the lavaan package in R ([Rosseel, 2012](#_ENREF_3)). The RMSEA and CFI indices for the three-factor model were 0.05 and .90, consistent with the literature ([Colins et al., 2015](#_ENREF_1)). Together, these findings indicate that the YPI is a valid instrument in our sample.

**References**

Colins, O. F., Andershed, H., & Pardini, D. A. (2015). Psychopathic traits as predictors of future criminality, intimate partner aggression, and substance use in young adult men. *Law and human behavior, 39*(6), 547.

Nichols, T. E., & Holmes, A. P. (2002). Nonparametric permutation tests for functional neuroimaging: a primer with examples. *Human brain mapping, 15*(1), 1-25.

Rosseel, Y. (2012). lavaan: An R package for structural equation modeling. *Journal of statistical software, 48*, 1-36.

Smith, S. M. (2002). Fast robust automated brain extraction. *Human brain mapping, 17*(3), 143-155.

Smith, S. M., Jenkinson, M., Johansen-Berg, H., Rueckert, D., Nichols, T. E., Mackay, C. E., & Behrens, T. E. (2006). Tract-based spatial statistics: voxelwise analysis of multi-subject diffusion data. *NeuroImage, 31*(4), 1487-1505.

Smith, S. M., & Nichols, T. E. (2009). Threshold-free cluster enhancement: addressing problems of smoothing, threshold dependence and localisation in cluster inference. *NeuroImage, 44*(1), 83-98.
